# Bioethics and human rights in nursing education: a report of a pedagogical experience*

**DOI:** 10.1590/1980-220X-REEUSP-2024-0384en

**Published:** 2025-07-07

**Authors:** Alessandra Lima Fontenele, Gabriele Pereira de Sena, Simone Luzia Fidelis de Oliveira, Dirce Bellezi Guilhem

**Affiliations:** 1 Universidade de Brasília, Faculdade de Ciências da Saúde, Programa de Pós-Graduação em Ciências da Saúde, Brasília, DF, Brazil.; 2 Fundação de Ensino e Pesquisa em Ciências da Saúde, Programa de Residência Uniprofissional de Enfermagem em Centro Cirúrgico. Brasília, DF, Brazil.; 3 Universidade de Brasília, Faculdade de Ciências da Saúde, Departamento de Enfermagem. Brasília, DF, Brazil.

**Keywords:** Bioethics, Human Rights, Teaching, Students, Nursing, Photography

## Abstract

**Objective:**

To describe a pedagogical experience with nursing students who used photographs as a teaching tool for teaching human rights.

**Method:**

This is a report of a pedagogical experience of a cross-sectional qualitative study based on narrative bioethics, which uses personal narratives as an analytical instrument to deepen the understanding of ethical problems, and on iconographic analysis, which investigates the deep meanings and contexts underlying the recorded photographs.

**Results:**

The photographs taken by “photographer students” portray real situations from everyday life in our society, highlighting issues such as poverty, social exclusion and government negligence, which, according to the participants themselves, evoke violations of several articles of the Universal Declaration of Human Rights.

**Final considerations:**

The pedagogical workshop used photography to engage students in reflections on human rights and social issues, promoting ethical and humanized training. Despite limitations, the study revealed a deficit in university education on human rights, but highlighted the potential of innovative methodologies to strengthen the critical and empathetic preparation of future professionals.

## INTRODUCTION

Photography is a powerful tool for historical knowledge, representing events and social groups, and acting as a pedagogical resource for analyzing frequently neglected topics. As a social experience, photography allows for varied interpretations, influenced by each individual’s cultural heritage. It also allows for the analysis of discourses that shape their interpretations based on the contexts in which they were created^(1,2)^.

Through photography, memories and emotions are immortalized, allowing social questions to be raised democratically^(2,3,4)^. In the field of human rights, photography denounces violations and struggles for dignity, immortalizing events such as wars and the achievement of rights^(5)^. These images contrast with the narrative of inalienable rights in the declarations, highlighting a world with countless violations^(5,6,7)^.

Thus, photography not only reflects reality, but also creates narratives that encourage critical reflection. In teaching human rights, it enables discussions about respect and fulfillment of these rights, especially among future healthcare professionals^(1,8,9)^. When capturing an image, a photographer constructs a discourse by deciding what to show, reflecting a subjective interpretation of reality^(9)^. Barthes^(10)^ sees a photographer as a narrator, whose choices influence observers’ perception.

This relationship between image and interpretation reflects the historical construction of human dignity, a central principle in human rights and bioethics, which is consolidated as an interdisciplinary field that studies life and its dimensions in the light of moral values and principles^(11,12)^. In this context, bioethical principlism, based on the principles of autonomy, beneficence, non-maleficence and justice, reinforces the need to guarantee equity and respect for human dignity, values also guaranteed by the Universal Declaration of Human Rights (UDHR)^(7,11,12)^.

Narrative bioethics, an emerging approach to bioethics and further explored by Tomás Moratalla^(13)^, complements this perspective by articulating moral principles with individual and collective stories that value the experience and context of each individual in ethical decision-making. In the teaching of human rights, this approach, associated with images, becomes a tool for awareness and critical analysis, encouraging reflections on dignity, justice, equity and bioethical principles, contributing to training citizens and healthcare professionals who are aware of and committed to ethics and human rights^(11,12,13,14)^.

In nursing, values such as dignity and respect for human rights are fundamental to ethical and humanized practice. The Brazilian National Curriculum Guidelines (In Portuguese, *Diretrizes Curriculares Nacionais* - DCNs) for undergraduate nursing courses emphasize the need for humanistic, critical and reflective training, preparing nurses to act with social responsibility and commitment to citizenship^(15)^. These guidelines highlight the importance of including content related to human rights, ethics and bioethics, enabling professionals to identify, address and prevent inequalities and discrimination in health^(16)^.

The inclusion of human rights and bioethics in nursing training ensures care based on respect for the autonomy, privacy and dignity of patients, fundamental principles supported by bioethical principlism and enriched by narrative bioethics^(11,12,13,14)^. In this way, training is not limited to technical-scientific development, but also strengthens nursing’s ethical, reflective and humanized commitment, in line with UDHR principles^(7,16)^.

Although the language of human rights is not yet widely used in the daily life of nursing, the principles established in the Code of Ethics for Nursing Professionals, such as well-being of patients and communities, respect, tolerance and honesty, directly reflect the rights guaranteed by UDHR, demonstrating the intersection between professional ethics and the fundamental values of bioethics and social justice^(7,17,18)^.

Therefore, considering the above, this study aimed to describe a pedagogical experience with nursing students who used photographs as a didactic tool for teaching human rights. This approach sought to strengthen future professionals’ commitment to building a more ethical and humane practice aligned with fundamental ethical and human rights principles.

## METHOD

### Study Design

This is a report of a pedagogical experience, with a qualitative and cross-sectional approach, carried out during the doctoral teaching internship in a graduate program in health sciences at a federal public university in Brazil. The study’s theoretical-methodological basis was supported by narrative bioethics and iconographic analysis, allowing a more sensitive and contextualized analysis of violations of UDHR principles^(7,13,14,19,20)^. To ensure methodological rigor, the study followed the COnsolidated criteria for REporting Qualitative research^(21)^.

### Study Place and Population

The study was conducted at a federal public university in Brazil, between March and July 2024, with students in the 5^th^ semester of an undergraduate nursing course, enrolled in the Bioethics and Legislation in Nursing (BLN) discipline. In total, 48 students were invited to participate in the “Photographic Pedagogical Workshop”, an activity proposed within the scope of the discipline. The BLN teaching plan highlights the obligation to address the theme of human rights and its importance for both healthcare and research. Therefore, the incorporation of photography as a pedagogical tool for critical reflection allowed students to explore, in a differentiated manner, the intersection between theory and practice, encouraging artistic skills and awareness of bioethical issues in healthcare, reinforcing its relevance for professional practice.

### Data Collection

The “Photographic Pedagogical Workshop” was organized in four stages, all designed to not compromise the progress of BLN. Its planning was carried out in conjunction with the professors in charge, aiming to ensure that the activities were in harmony with the academic schedule planned for the semester.

The first two stages consisted of theoretical classes that addressed fundamental concepts, history and guidelines on human rights, as well as the role of photography in defending these rights, highlighting it as a tool for denunciation and social transformation. These meetings addressed the theme of human rights, as provided for in the course’s teaching plan, providing students with a solid theoretical foundation and a critical understanding that prepared them for the subsequent moments.

The third moment was dedicated to a voluntary practical activity, which consisted of sending an unpublished photograph illustrating the violation of some article of UDHR^(7)^. Violations could address issues such as integrity (physical, psychological or patrimonial), freedom, security, social, civil and political rights, life, and violation of environmental rights. However, the specification of the type of violation would depend on the record chosen by the academic, which means that not all forms of violations could be considered in the exercise^(7)^.

Students were instructed to capture the images (in color or black and white) with cell phones, due to their ease of use. For students who chose to include individuals in images, two copies of the Term of Assignment of Use of Image for Scientific Purposes were made available: one for the model and another to be given to professors for storage. They were instructed to post their photographs, with a title, the cell phone model used, and a caption that included UDHR article^(7)^ that had been violated (criteria for including the images in the study), in the virtual classroom of the course linked to their enrollment and to the university’s institutional email. Only the professors and the research team were allowed to manage the virtual classroom content, which ensured image confidentiality and organization. Furthermore, this activity was optional and non-assessed, ensuring that failure to submit the images would not result in academic penalties.

Finally, the fourth part of the workshop was dedicated to discussion, in class and in person, of the photographs taken by students. During this stage, images were presented together with their iconographic analysis. This allowed all the students to carry out a critical analysis of the photographs taken and the violated articles, in order to enable a later critical analysis.

### Data Analysis and Processing

In this study, data analysis was based on Panofsky’s^(19)^ iconographic analysis and Tomás Moratalla’s^(13)^ narrative bioethics. Panofsky’s approach involved three stages: pre-iconographic, which examines images’ visual elements; iconographic, which interprets the meanings and symbols contained in them; and iconological, which contextualizes the elements in their historical and cultural setting. Narrative bioethics complemented this approach by focusing on the experiences underlying the images, helping to interpret the narratives and connections with the observed violations of UDHR^(7,13,14,19,20)^.

Tomás Moratalla’s^(13)^ perspective, applied in this study, highlights narratives as a pedagogical tool in bioethics, allowing students to understand ethical dilemmas through concrete experiences and stories represented in images. This vision, by integrating narrative analysis with the ethical challenges of professional practice, strengthened the connection between bioethics, human rights and social reality, preparing students to deal with complex situations in healthcare^(11,12,13,14)^.

### Ethical Aspects

This study followed the ethical principles of Resolution 466/2012^(22)^ of the Brazilian National Health Council, and was initiated only after approval by the Research Ethics Committee of the School of Health Sciences of *Universidade de Brasília*, Consolidated Opinion 6.312.687, 09/20/2023. All students were informed about the research and agreed to participate voluntarily, by signing the Informed Consent Form.

To ensure anonymity, participants were identified as “photographer student (PS)”, followed by a numerical code, assigned according to the chronological order in which photographs were received (e.g., PS01, PS02, etc.). Participants who did not send photographs were identified as “observer student (OS)”, followed by a numerical code starting with the number 29, in continuity with the numbering of workshop participants and totaling the number of students enrolled in the course (e.g., OS29, OS30, etc.).

## RESULTS

Forty-eight students enrolled in the BLN course participated in the study. Of these, 28 submitted photographs for the practical activity in the third moment. However, two images were excluded because they did not meet established criteria, resulting in a final sample of 26 photographs for analysis (pre-iconographic and iconographic interpretation). The students who did not submit photographs, choosing to participate only in the theoretical classes (first and second moments) and the discussion in the fourth moment, shared their narratives about the images of their colleagues.

Preliminary analysis allowed photographs to be categorized into three themes: (1) Garbage as a symbol of human interference, representing waste as signs of abandonment and neglect; (2) Urbanization as a point of social reflection, highlighting the contradictions between modernization and marginalization; and (3) Man as the center of inequalities, highlighting the vulnerabilities and impacts of social injustices on individuals. These images were organized according to the chronological order in which they were received, which justifies the absence of a linear sequence within the categories.

**Figure 1:**
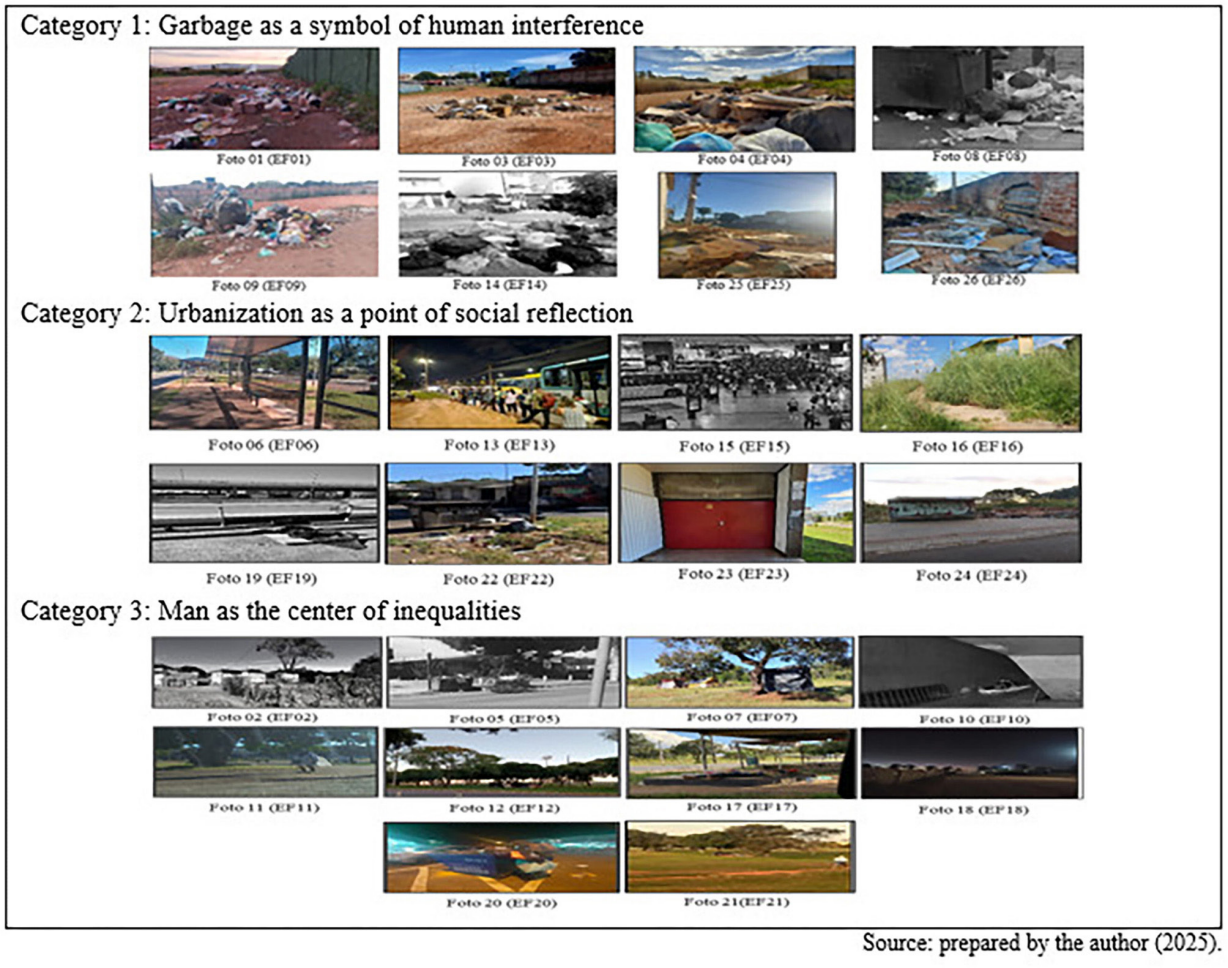
Compilation of photographs taken by students during the practical activity – Brasília, FD, Brazil, 2025.

As illustrated in Figure 1, the photographs taken by PSs portray poverty, social exclusion and government neglect, highlighting several human rights violations. After preliminary analysis, it was observed that violations related to freedom, social, civil and political rights, the right to life and the environment, as established in UDHR, predominated^(7)^.

### Pre-Iconographic Analysis

**Figure 2:**
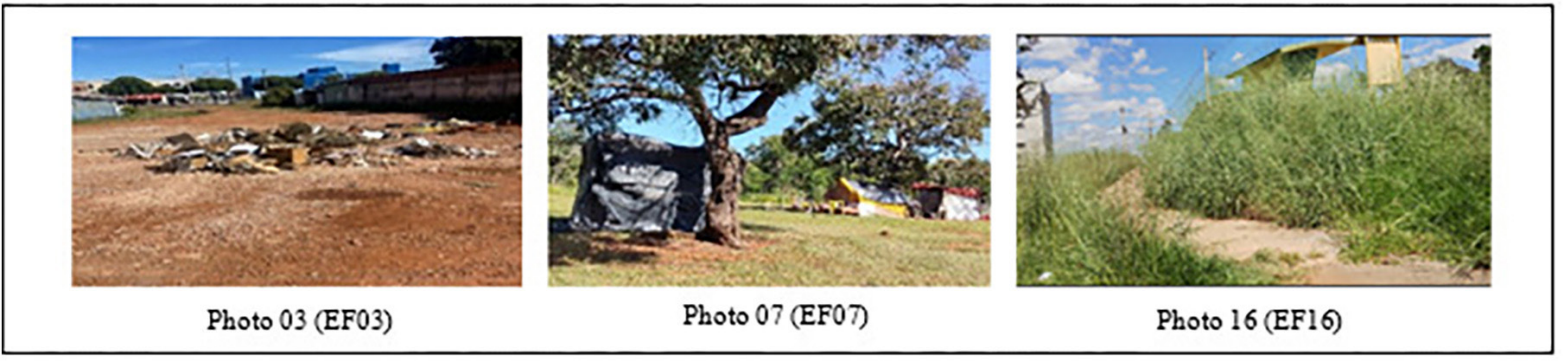
Elements of degradation observed in photographs – Brasília, FD, Brazil, 2025.

Preliminary analysis of photographs focused on the objective observation of visible and recurring elements in images, such as shapes, colors, figures and the composition of elements, without attributing interpretations or meanings^(19,20)^. In view of this, it was possible to identify the constant presence of degrading elements in the environment, such as garbage, debris, graffiti and tall vegetation. In some of these images, improvised shelters were recorded, located in unsuitable areas and exposed to risks (Figure 2).

**Figure 3:**
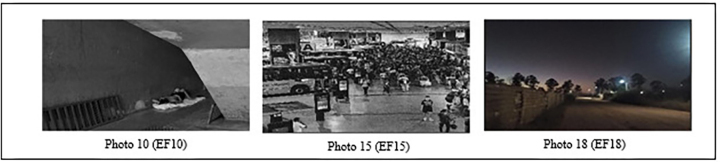
Human figures observed in photographs – Brasília, FD, Brazil, 2025.

On the other hand, although less frequent, some photographs revealed human figures in everyday situations, such as individuals waiting for public transportation, holding garbage bags, and receiving healthcare (Figure 3). The photographic composition of these images also stood out, featuring cooler colors, including black and white options, which contrast with the warm colors predominant in other photographs. Another notable aspect of the composition concerns the imaginary lines that, “drawn” by PSs during image capture, guided the observer’s vision towards the highlighted elements.

This aesthetic variation and the elements present in photographs enriched the visual narrative, providing a more complex and multifaceted understanding of urban life vulnerable to violations of UDHR^(7)^, as captured by PSs. Furthermore, this aesthetic diversity contributed to structuring the photographic narrative, facilitating the iconographic interpretation of images.

### Iconographic Interpretation

Iconographic identification involved the analysis of images, attributing meanings to previously identified visual elements. The main objective of this stage was to interpret the symbols, figures and compositions observed, placing them in their social, religious, cultural or historical contexts^(19,20)^. In the case of photographs recorded by PSs, iconographic identification allowed us to explore how the captured visual elements reflect issues related to human rights violations and social vulnerability.

By highlighting elements in the photographs that demonstrate environmental degradation, for instance, PSs indicate the lack of maintenance and care in collective living spaces, symbolizing the neglect of areas that are generally occupied by vulnerable populations. This scenario of abandonment can be understood as a reflection of the marginalization faced by these communities, who often live without access to basic services and quality infrastructure.

**Figure 4:**
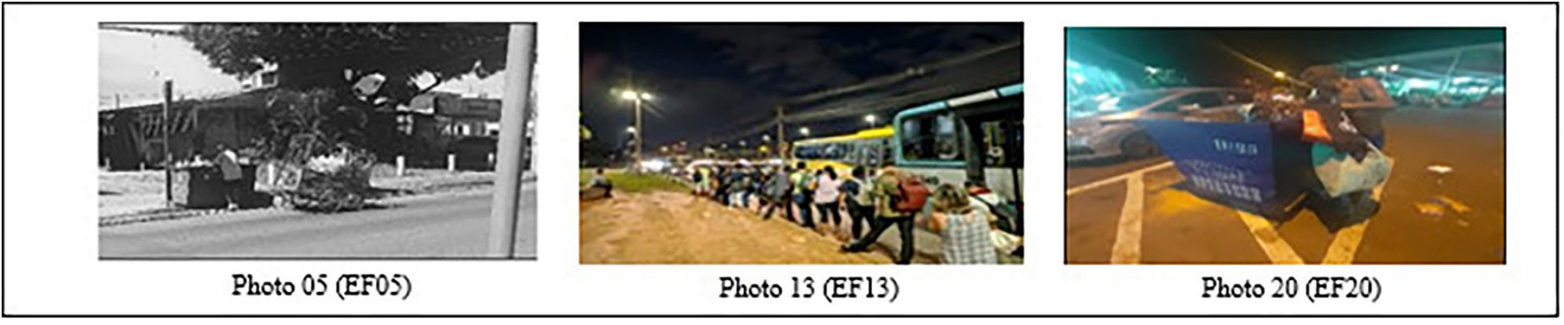
Photographs of everyday scenes of social vulnerability – Brasília, FD, Brazil, 2025.

From another perspective, photographs that include human figures, despite depicting everyday scenes, also reveal layers of social vulnerability and human rights violations when observed in depth (Figure 4). These images capture not only individuals’ immediate actions, but suggest contexts of precariousness and helplessness that transcend the obvious, referring to structural issues and the absence of basic guarantees.

The scene of individuals waiting for public transport, for instance, can be interpreted as a symbol of the lack of access to an efficient, safe and adequate transport system, evidencing an indirect violation of the right to mobility and freedom of movement^(7)^. This limitation not only hinders movement, but also reflects the lack of urban infrastructure necessary to guarantee dignity and well-being for communities.

Another symbolism presented in the photographs is the representation of people carrying garbage bags, an act that, in addition to highlighting the relationship between residents and waste management, brings to mind the idea of garbage as a source of food. This reality reflects the abandonment of fundamental rights, in addition to illustrating dehumanization and food insecurity, which are violations of the right to a healthy environment, life and dignity^(7)^.

In iconographic interpretation, it was possible to perceive inequality and social exclusion in the images of PSs even before identifying the violated articles of UDHR^(7)^. Photographic compositions, carefully prepared by them, combined with their knowledge of the articles of the declaration, a topic addressed in the initial stages of the workshop, facilitated the identification of the symbolism present in images and the preliminary mapping of the rights violated even before they were presented in their narratives.

### Iconological Interpretation and Narrative Bioethics

Iconological interpretation goes beyond the simple visual and symbolic description of images; it seeks to explore the ideas, values and deep meanings of elements within the historical and social context in which the work was created^(19,20)^. Narrative bioethics, in turn, complemented this analysis by enriching the understanding of works that deal with themes such as life and the human condition, providing a deeper insight into the ethical and emotional issues represented^(13,14)^.

In the case of photographs taken by PSs, this integrated approach made it possible to explore not only the images’ visual and technical aspects, but also the personal and collective narratives they carry. In the bioethical context, these violations challenge principlism principles, compromising autonomy, justice and beneficence in healthcare and human dignity^(11,12)^. Narrative bioethics complements this perspective by transforming images into visual narratives that expose these inequalities and ethical dilemmas^(13,14)^. This process encourages empathy and critical reflection, helping students understand the impact of human rights violations on nursing care and reinforcing the importance of a practice based on ethics, equity and human dignity.

The inclusion of these emotions in the recording directly influenced the impact that each student wanted to convey to their observer. The “tactile” elements of photography and the symbolism present in the composition of images intertwined with the ideas of human rights and bioethical principles, shaping the perception and interpretation of all the actors who analyzed them. In this way, each analysis was enriched by these elements, which, together, broadened the experience and understanding of the visual works.

By discussing the emotions and reflections that emerged during capture, students were able to articulate how these images represent fragments of the daily violations in our society. Their verbalizations enriched the understanding of the photographs and articles they evoke, promoting a discussion that included OSs’ perceptions, who did not take pictures but were able to identify other violations that were less evident to the photographer.

During the analyses, PSs highlighted that they often do not notice the violations of rights that occur daily with the people around them, due to the normalization of these situations in various contexts. This aspect was also highlighted by OSs, who acknowledged that they often do not notice the violations around them as vividly as in photographs. Many of these students even reported that because they do not notice such explicit violations in their contexts, they chose not to send their own images:

*I was interested, but I didn’t have time to look for anything beyond what was already being photographed by other colleagues. However, I realize that, regardless of how explicit they are, violations are always present around us!* (OS29).

**Figure 5:**
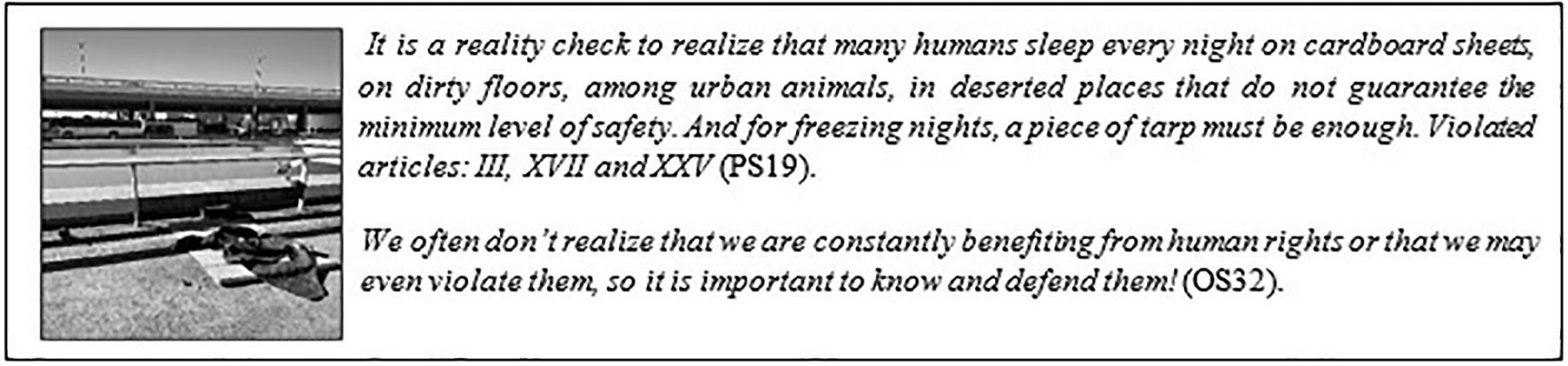
Photograph 19 and students’ narratives – Brasília, FD, Brazil, 2025.

The OS not only recognized and understood the symbolism behind the photographs, but also expanded interpretations, bringing new perspectives that had not been previously mentioned. This dialogue demonstrates a deeper understanding of the social issues at hand, allowing all students, regardless of their participation, to engage with the issues raised by photographs (Figure 5).

When analyzing the social role of images, all students pointed out that both the photographs they took and those presented during the workshop play a fundamental role as tools for critical communication and social transformation in different contexts. For students, images are not only representations of reality, but also powerful instruments for provoking reflection, generating debate and questioning established norms:

*It is through photography that we can highlight different realities and advance important discussions for society* (OS32).

*Photography is more than just a portrayal of important moments; it can be a form of criticism and social denunciation, pointing out rights that are violated and realities that need to be changed* (OS40).

With regard to photographs that record violations of rights provided for in UDHR^(7)^, their great potential to raise public awareness, influence attitudes and even inspire significant social change is observed. This impact is particularly relevant for students, as they pointed out, since they are future healthcare professionals and, therefore, will be inserted in vulnerable work contexts or in direct contact with populations at risk.

It is clear that, for all students, recording violations of articles of UDHR^(7)^, as well as understanding photography as a tool for social criticism, is not something impossible or difficult to achieve, since situations of poverty, social exclusion and government neglect are constantly present in our society, and visualizing these violations can foster the development of critical thinking. However, they emphasize that identifying these violations is clearer with prior knowledge of theoretical concepts, especially in the academic context:

*From the approach to the subject of human rights and bioethics, I feel that my perspective and critical sense have improved in relation to the beginning of the discipline. If it were addressed more broadly within the university, students would have a more humane education* (OS35).

This foundation allows for a more critical and in-depth reading of reality, facilitating the perception of structural inequalities and rights that are being disrespected. In the context of health, as they point out, this differentiated education is essential, as it would prepare them to identify and address inequalities in access to care as well as discrimination that may occur in health systems. By integrating this approach into nursing training, the importance of a practice based on equity, respect for human rights, and the promotion of fairer and more inclusive care is reinforced.

Subsequently, in the discussion section, the implications of photography as a pedagogical tool in human rights education will be addressed in conjunction with narrative bioethics and bioethical principlism. Its impacts on critical and ethical reflection will be analyzed, highlighting social violations and their reflections on nursing practice.

## DISCUSSION

Susan Sontag (2004) argues that photography transforms events into consumable objects, creating limited and subjective narratives of reality. She sees photographs as constructions that can simplify the complexity of the original event, making observers’ active analysis essential to its understanding^(23)^. Supporting this idea, Roland Barthes^(10)^ presents photography as a form of narrative that documents and creates a personal experience^(10)^.

The union of Sontag’s and Barthes’ ideas allows for a profound reflection on image, reality and interpretation. Sontag sees photography as a form of consumption that creates restricted visual narratives, while Barthes highlights its intimate dimension, in which context and emotional detail create a unique experience, transforming the relationship between photographer and observer^(10,23)^. Thus, photography, as a tool for transformation, aligns with the concepts of narrative bioethics, as it not only records a reality, but also shapes unique ethical and emotional responses.

Narrative bioethics values this ability to feel and interpret other people’s experiences, allowing photography to transcend its role as a mirror of reality, becoming a space for dialogue and empathy in human issues^(13,14)^. Applying Erwin Panofsky’s iconological analysis, which examines images in their historical and cultural contexts, we see that they reveal deep layers of meaning. These layers are crucial to fostering empathy and ethical understanding, enriching both the experience of photographers and observers^(19,20)^.

These concepts help to understand how nursing students who participated in the workshop perceive human rights violations in our society, highlighting photography as a social critique. They understood that it not only documents, but also engages and provokes empathy, addressing injustice in a way that articulates discourses about humanity and its vulnerabilities, functioning as a link between the present and social transformation^(1,3,4,5,6)^.

From the perspective of bioethical principlism, this approach reinforces in nursing students the importance of the principles of autonomy, justice, beneficence and non-maleficence in health. By exposing inequalities and encouraging critical reflection, photography broadens the perception of ethical responsibility in the defense of human rights. Thus, image analysis raises awareness of the challenges faced by vulnerable populations, strengthening the need for professional practice based on equity, respect and collective well-being^(11,12)^.

In the static image format, photography represents a close reality, but, as Secco (2021) points out, it remains subject to external influences from both photographers and observers. When enriched by narratives, photography transcends its documentary role and can become an essential academic instrument for promoting debates and deep reflections on social issues and human rights violations, topics that are interconnected with daily nursing practices. Moreover, this approach strengthens student critical training, encouraging more humanized care that is ethically committed to patient equity and dignity^(11,12)^.

Thus, the encounter between narrative bioethics and photography occurs when using nursing students’ narratives and experiences in capturing images as instruments to encourage critical reflection and ethical deliberation in healthcare. This process of integrating narrative with the act of photography in teaching goes beyond the simple transmission of knowledge, engaging nursing students in an in-depth and active analysis of ethical and social issues, promoting a more holistic and responsible understanding of the complexities involved in healthcare practice^(13,14)^.

Studies with participants from different areas and levels of education show that photography is an effective tool for raising awareness about sensitive issues, constructing image narratives that deconstruct mechanistic practices and broaden critical understanding^(10,19,23,24,25)^. In this way, this approach strengthens the training of students, enabling them to identify human rights violations and reflect on the ethical conduct to be adopted in professional nursing practice^(15,16,17,18,25)^. Furthermore, it promotes more humanized care, reaffirming nursing’s commitment to social justice, ethical principles and patient dignity^(11,12)^. In higher education, it is vital to create a culture of human rights, as indicated by DCNs and the Brazilian National Policy on Human Rights Education^(15,26)^, which is essential for defending the most vulnerable. This education should offer not only technical knowledge, but also develop critical and ethical skills, preparing future professionals to act responsibly and thoughtfully^(17,26,27 28)^.

Integrating human rights education, in alignment with ethical principles, into the curriculum prepares the individual as an agent of change open to dialogue and committed to dignity and equity^(11,12,17,18,27,28)^. For this learning to be effective, it is essential that education goes beyond theory, using practical activities such as photography. This method not only encourages deep reflection on the ethical and social implications of professional practice, but also motivates students to actively engage in interactive activities, promoting engaging and critical learning^(20,27,28)^.

Therefore, it is clear that health students, especially nursing students, must receive technical training and content that integrates theory and care practice, allowing them to be included in the routines and protocols of healthcare institutions^(25,26,27,28)^. The inclusion of cross-cutting themes in human rights helps to train professionals who are capable and aware of how to act ethically and with respect for patient dignity and vulnerability.

Despite the small sample and the application in a single course, the main shortcoming of this study was the absence of photographs that represented a greater diversity of human rights violations. This limitation possibly stems from the low familiarity of nursing students with the theme of human rights throughout their undergraduate studies, which may have resulted in the invisibility of violations present in their daily lives. This scenario highlights the need to broaden the approach to human rights in nursing education, integrating UDHR in an interdisciplinary manner into the theoretical and practical curriculum. Including this theme in a more structured way would not only contribute to the development of a critical and reflective vision of students, but would also strengthen their professional performance in recognizing and confronting social injustices and health inequalities.

## CONCLUSION

The pedagogical workshop developed in this study was a meaningful experience, allowing students to engage in both the capture and critical analysis of images. The use of photographs as a didactic tool for teaching human rights encouraged reflection on social issues and human rights, broadening participants’ understanding of the ethical implications for their future professional practice.

Furthermore, this pedagogical experience can be used as a tool to identify students’ weaknesses regarding their perception and understanding of violations of UDHR, allowing professors to deepen and expand the debate. The lack of an integrated approach between bioethics, nursing and human rights limits nurses’ critical and ethical training.

Therefore, the association between pedagogical practices, such as narrative bioethics, principlism, human rights and the use of photography, as a methodological tool for the integration and application of these frameworks was essential. This fact contributed to the preparation of future empathetic professionals, sensitive to violations, with ethical awareness about dignity and vulnerabilities in the face of violations of UDHR, experienced in the daily professional life of nursing.

## Data Availability

The dataset supporting the findings of this study is not publicly available due to the presence of sensitive information and the need to preserve participant confidentiality. The photographs and other materials produced during the pedagogical workshop are stored in an institutional academic repository with restricted access to the research team, as approved by the Research Ethics Committee of the Faculty of Health Sciences at the University of Brasília (UnB) (Approval Report No. 6.312.687, dated September 20, 2023). All relevant information necessary for understanding the findings is described within the body of the article.
