# Supplementary material for: Bioethics and human rights in nursing education: a report of a pedagogical experience
Source: Rev Esc Enferm USP. 2025 Jul 7;59:e20240384. doi: 10.1590/1980-220X-REEUSP-2024-0384en (PMC12245390; doi:10.1590/1980-220X-REEUSP-2024-0384en)
Supplement: Translation

# Translation: Bioética e direitos humanos no ensino de enfermagem: um relato de experiência pedagógica*

**DOI:** 10.1590/1980-220X-REEUSP-2024-0384pt

**Authors:** Alessandra Lima Fontenele, Gabriele Pereira de Sena, Simone Luzia Fidelis de Oliveira, Dirce Bellezi Guilhem

**Affiliations:** 1 Universidade de Brasília, Faculdade de Ciências da Saúde, Programa de Pós-Graduação em Ciências da Saúde, Brasília, DF, Brasil.; 2 Fundação de Ensino e Pesquisa em Ciências da Saúde, Programa de Residência Uniprofissional de Enfermagem em Centro Cirúrgico. Brasília, DF, Brasil.; 3 Universidade de Brasília, Faculdade de Ciências da Saúde, Departamento de Enfermagem. Brasília, DF, Brasil.

**Keywords:** Bioética, Direitos Humanos, Ensino, Estudantes de Enfermagem, Fotografia

## Abstract

**Objetivo:**

Descrever uma experiência pedagógica com estudantes de enfermagem que usaram fotografias como ferramenta didática para o ensino de direitos humanos.

**Método:**

Trata-se do relato de experiência pedagógica de estudo qualitativo transversal fundamentado na bioética narrativa, que utiliza narrativas pessoais como instrumento analítico para aprofundar a compreensão de problemas éticos, e na análise iconográfica, que investiga os significados profundos e os contextos subjacentes às fotografias registradas.

**Resultados:**

As fotografias capturadas pelos “estudantes fotógrafos” retratam situações reais do cotidiano de nossa sociedade evidenciando questões como pobreza, exclusão social e negligência governamental, as quais, segundo os próprios participantes, evocam violações de diversos artigos da Declaração Universal de Direitos Humanos.

**Considerações finais:**

A oficina pedagógica usou a fotografia para engajar estudantes em reflexões sobre direitos humanos e questões sociais, promovendo formação ética e humanizada. Apesar de limitações, o estudo revelou um déficit na educação universitária em direitos humanos, mas destacou o potencial de metodologias inovadoras para fortalecer a preparação crítica e empática dos futuros profissionais.

## INTRODUÇÃO

A fotografia é uma ferramenta poderosa para o conhecimento histórico, representando eventos e grupos sociais, e atuando como recurso pedagógico para análise de temas frequentemente negligenciados. Como uma experiência social, a fotografia permite interpretações variadas, influenciadas pelo patrimônio cultural de cada indivíduo. Ela também possibilita a análise dos discursos que moldam suas interpretações a partir dos contextos em que foram criadas^(1,2)^.

Por meio da fotografia, memórias e emoções são eternizadas, permitindo questionamentos sociais de forma democrática^(2-4)^. No campo dos direitos humanos, a fotografia denuncia violações e lutas por dignidade, imortalizando eventos como guerras e conquistas de direitos^(5)^. Essas imagens contrastam com a narrativa de direitos inalienáveis das declarações, evidenciando um mundo com inúmeras violações^(5#x2013;7)^.

Assim, a fotografia não apenas reflete a realidade, mas também cria narrativas que incentivam reflexões críticas. No ensino de direitos humanos, ela possibilita discussões sobre respeito e cumprimento desses direitos, especialmente entre futuros profissionais de saúde^(1,8,9)^. Ao capturar uma imagem, o fotógrafo constrói um discurso ao decidir o que mostrar, refletindo uma interpretação subjetiva da realidade^(9)^. Barthes^(10)^ vê o fotógrafo como um narrador, cujas escolhas influenciam a percepção do observador.

Essa relação entre imagem e interpretação reflete a construção histórica da dignidade humana, princípio central nos direitos humanos e na bioética, que é consolidada como um campo interdisciplinar que estuda a vida e suas dimensões à luz de valores e princípios morais^(11,12)^. Nesse contexto, o principialismo bioético, baseado nos princípios de autonomia, beneficência, não maleficência e justiça, reforça a necessidade de garantir equidade e respeito à dignidade humana, valores também assegurados pela Declaração Universal dos Direitos Humanos (DUDH)^(7,11,12)^.

A bioética narrativa, uma abordagem emergente da bioética e aprofundada por Tomás Moratalla^(13)^, complementa essa perspectiva ao articular princípios morais com as histórias individuais e coletivas que valorizam a experiência e o contexto de cada indivíduo na tomada de decisão ética. No ensino de direitos humanos, essa abordagem, associada às imagens, torna-se uma ferramenta de sensibilização e análise crítica, incentivando reflexões sobre dignidade, justiça, equidade e princípios bioéticos, contribuindo para a formação de cidadãos e profissionais de saúde conscientes e comprometidos com a ética e os direitos humanos^(11#x2013;14)^.

Na enfermagem, valores como dignidade e respeito aos direitos humanos são fundamentais para uma prática ética e humanizada. As Diretrizes Curriculares Nacionais (DCNs) para os cursos de graduação em enfermagem enfatizam a necessidade de uma formação humanista, crítica e reflexiva, preparando enfermeiros para atuar com responsabilidade social e compromisso com a cidadania^(15)^. Essas diretrizes ressaltam a importância da inclusão de conteúdos relacionados a direitos humanos, ética e bioética, capacitando os profissionais para identificar, enfrentar e prevenir desigualdades e discriminações na saúde^(16)^.

A inclusão dos direitos humanos e da bioética na formação do enfermeiro assegura um cuidado pautado no respeito à autonomia, privacidade e dignidade dos pacientes, princípios fundamentais sustentados pelo principialismo bioético e enriquecidos pela bioética narrativa^(11#x2013;14)^. Dessa forma, a formação não se limita ao desenvolvimento técnico-científico, mas também fortalece o compromisso ético, reflexivo e humanizado da profissão, em consonância com os princípios da DUDH^(7,16)^.

Embora a linguagem dos direitos humanos ainda não seja amplamente utilizada no cotidiano da profissão, os princípios estabelecidos no Código de Ética dos Profissionais de Enfermagem, como o bem-estar do paciente e da comunidade, respeito, tolerância e honestidade, refletem diretamente os direitos assegurados pela DUDH, demonstrando a interseção entre a ética profissional e os valores fundamentais da bioética e da justiça social^(7,17,18)^.

Logo, diante do exposto, este estudo teve como objetivo descrever uma experiência pedagógica com estudantes de enfermagem que usaram fotografias como ferramenta didática para o ensino de direitos humanos. Essa abordagem buscou fortalecer o compromisso dos futuros profissionais com a construção de uma prática mais ética e humana alinhada aos princípios fundamentais éticos e dos direitos humanos.

## MÉTODO

### Tipo de Estudo

Trata-se de relato de experiência pedagógica, com abordagem qualitativa e de corte transversal, realizado durante o estágio de docência de doutorado em um programa de pós-graduação em ciências da saúde de uma universidade pública federal do Brasil. A base teórico-metodológica do estudo apoiou-se na bioética narrativa e na análise iconográfica, permitindo uma análise mais sensível e contextualizada sobre as violações dos princípios da DUDH^(7,13,14,19,20)^. Para garantir o rigor metodológico, o estudo seguiu os critérios do *COnsolidated criteria for REporting Qualitative research* ^(21)^.

### Local e População do Estudo

O estudo foi realizado em universidade pública federal do Brasil, entre março e julho de 2024, com estudantes do 5º semestre do curso de graduação em enfermagem, matriculados na disciplina de Bioética e Legislação em Enfermagem (BLE). Ao todo, 48 estudantes foram convidados a participar da “Oficina Pedagógica Fotográfica”, atividade proposta no âmbito da disciplina. O plano de ensino da BLE destaca a obrigatoriedade de abordar a temática dos direitos humanos e a sua importância tanto para a assistência quanto para a pesquisa em saúde. Logo, a incorporação da fotografia como ferramenta pedagógica de reflexão crítica permitiu aos estudantes explorar, de forma diferenciada, a interseção entre teoria e prática, estimulando habilidades artísticas e a conscientização sobre questões bioéticas na saúde, reforçando sua relevância para a prática profissional.

### Coleta de Dados

A “Oficina Pedagógica Fotográfica” foi organizada em quatro etapas, todas concebidas para não comprometer o andamento da disciplina de BLE. Seu planejamento foi realizado em conjunto com as professoras responsáveis, visando assegurar que as atividades estivessem em harmonia com o cronograma acadêmico previsto para o semestre.

As duas primeiras etapas consistiram em aulas teóricas que abordaram os conceitos fundamentais, a história e as diretrizes sobre direitos humanos, além do papel da fotografia na defesa desses direitos, destacando-a como ferramenta de denúncia e transformação social. Esses encontros abordaram a temática dos direitos humanos, conforme previsto no plano de ensino da disciplina, proporcionando aos estudantes uma base teórica sólida e um entendimento crítico que os preparou para os momentos subsequentes.

O terceiro momento foi destinado a uma atividade prática voluntária, que consistia no envio de uma fotografia inédita ilustrando a violação de algum artigo da DUDH^(7)^. As violações poderiam abordar temas como integridade (física, psíquica ou patrimonial), liberdade, segurança, direitos sociais, civis e políticos, vida, e violação de direitos voltados ao meio ambiente. Entretanto, a especificação do tipo de violação dependeria do registro escolhido pelo acadêmico, o que significa que nem todas as formas de violações poderiam ser contempladas no exercício^(7)^.

Os alunos foram orientados a capturar as imagens (coloridas ou preto e branco) com celulares, devido à facilidade de uso. Para os estudantes que optassem por incluir indivíduos nas imagens, foram disponibilizadas duas vias do Termo de Cessão de Uso de Imagem para Fins Científicos: uma para o modelo e outra a ser entregue às professoras, para o armazenamento. Estes foram orientados a postar suas fotografias, com título, o modelo de celular utilizado e uma legenda que incluísse o artigo da DUDH^(7)^ violado (critérios de inclusão das imagens no estudo), na sala virtual da disciplina vinculada à matrícula e ao e-mail institucional universitário. Somente as professoras e a equipe de pesquisa tinham permissão para gerenciar o conteúdo da sala virtual, o que assegurou a confidencialidade e a organização das imagens. Além disso, esta atividade foi opcional e não avaliativa, garantindo que a não entrega não resultasse em penalidades acadêmicas.

Por fim, o quarto momento da oficina foi dedicado à discussão, em sala de aula e de forma presencial, das fotografias registradas pelos estudantes. Durante essa etapa, as imagens foram apresentadas juntamente com sua análise iconográfica. Isso permitiu que todos os estudantes realizassem uma análise crítica das fotografias registradas e dos artigos violados, a fim de possibilitar uma análise crítica posterior.

### Análise e Tratamento dos Dados

Neste estudo, a análise dos dados foi baseada na análise iconográfica de Panofsky^(19)^ e na bioética narrativa de Tomás Moratalla^(13)^. A abordagem de Panofsky envolveu três etapas: pré-iconográfica, que examina os elementos visuais das imagens; iconográfica, que interpreta os significados e símbolos nelas contidos; e iconológica, que contextualiza os elementos em seu cenário histórico e cultural. A bioética narrativa complementou essa abordagem ao focar as experiências subjacentes às imagens, ajudando a interpretar as narrativas e as conexões com as violações observadas da DUDH^(7,13,14,19,20)^.

A perspectiva de Tomás Moratalla^(13)^, aplicada neste estudo, destaca as narrativas como ferramenta pedagógica na bioética, permitindo que os estudantes compreendessem os dilemas éticos por meio de experiências concretas e histórias representadas nas imagens. Essa visão ao integrar a análise narrativa, junto aos desafios éticos da prática profissional, fortaleceu a conexão entre bioética, direitos humanos e realidade social, preparando os estudantes para lidar com situações complexas na assistência^(11#x2013;14)^.

### Aspectos Éticos

Este estudo seguiu os princípios éticos da Resolução n^o^ 466/2012^(22)^ do Conselho Nacional de Saúde, e foi iniciado apenas após a aprovação pelo Comitê de Ética em Pesquisa da Faculdade de Ciências da Saúde da Universidade de Brasília, Parecer Consubstanciado nº 6.312.687, 20/09/2023. Todos os estudantes foram informados sobre a pesquisa e concordaram em participar voluntariamente, por meio da assinatura do Termo de Consentimento Livre e Esclarecido.

Para garantir o anonimato, os participantes foram identificados como “estudante fotógrafo (EF)”, seguido de um código numérico, atribuído conforme a ordem cronológica de recebimento das fotografias (por exemplo: EF01, EF02, etc.). Os participantes que não enviaram fotografias foram identificados como “estudante observador (EO)”, seguidos de um código numérico iniciado a partir do número 29, em continuidade à numeração dos participantes da oficina e totalizando o número de estudantes matriculados na disciplina (por exemplo: EO29, EO30, etc.).

## RESULTADOS

Participaram do estudo 48 estudantes matriculados na disciplina de BLE. Entre esses, 28 enviaram fotografias para a atividade prática do terceiro momento. No entanto, duas imagens foram excluídas por não atenderem aos critérios estabelecidos, resultando em uma amostra final de 26 fotografias para análise (pré-iconográfica e interpretação iconográfica). Os estudantes que não realizaram o envio das fotografias, optando por participar apenas das aulas teóricas (primeiro e segundo momentos) e da discussão no quarto momento, compartilharam suas narrativas sobre as imagens de seus colegas.

A análise preliminar permitiu a categorização das fotografias em três temas: (1) *O lixo como símbolo da interferência*, representando os resíduos como marcas de abandono e descaso; (2) *A urbanização como ponto de reflexão social*, destacando as contradições entre modernização e marginalização; e (3) *O homem como centro das desigualdades*, evidenciando as vulnerabilidades e os impactos das injustiças sociais sobre os indivíduos. Essas imagens foram organizadas conforme a ordem cronológica de recebimento, o que justifica a ausência de uma sequência linear dentro das categorias.

Conforme ilustrado na Figura 1, as fotografias capturadas pelos EFs retratam pobreza, exclusão social e negligência governamental, evidenciando diversas violações de direitos humanos. Após a análise preliminar, observou-se que predominaram violações ligadas à liberdade, direitos sociais, civis e políticos, direito à vida e meio ambiente, conforme estabelecido na DUDH^(7)^.

**Figura 1:** Compilado das fotografias registradas pelos estudantes durante a atividade prática – Brasília, DF, Brasil, 2025.

### Análise Pré-Iconográfica

A análise preliminar das fotografias concentrou-se na observação objetiva de elementos visíveis e recorrentes nas imagens, como formas, cores, figuras e a composição dos elementos, sem atribuir interpretações ou significados^(19,20)^. Diante disso, foi possível identificar a presença constante de elementos degradantes no ambiente, como lixo, entulho, pichações e vegetação alta. Em algumas dessas imagens, foram registrados abrigos improvisados, localizados em áreas inadequadas e expostas a riscos (Figura 2).

**Figura 2:** Elementos de degradação observados nas fotografias – Brasília, DF, Brasil, 2025.

Em contrapartida, embora menos frequentes, algumas fotografias revelaram figuras humanas em situações cotidianas, como indivíduos à espera de transporte público, segurando sacolas de lixo e recebendo cuidados de saúde (Figura 3). A composição fotográfica dessas imagens também se destacou, apresentando cores mais frias, incluindo opções em preto e branco, que contrastam com as cores quentes predominantes nas demais fotografias. Outro aspecto notável na composição diz respeito às linhas imaginárias que, “desenhadas” pelos EFs durante a captura da imagem, orientavam a visão do observador em direção aos elementos de destaque.

**Figura 3:** Figuras humanas observadas nas fotografias – Brasília, DF, Brasil, 2025.

Essa variação estética e os elementos presentes nas fotografias enriqueceram a narrativa visual, proporcionando uma compreensão mais complexa e multifacetada da vida urbana vulnerável às violações da DUDH^(7)^, conforme capturado pelos EFs. Além disso, essa diversidade estética contribuiu para a estruturação da narrativa fotográfica, facilitando a interpretação iconográfica das imagens.

### Interpretação Iconográfica

A identificação iconográfica envolveu a análise das imagens, atribuindo significados aos elementos visuais previamente identificados. O objetivo central desta etapa foi interpretar os símbolos, figuras e composições observadas, situando-os em seus contextos sociais, religiosos, culturais ou históricos^(19,20)^. No caso das fotografias registradas pelos EFs, a identificação iconográfica permitiu explorar como os elementos visuais capturados refletem questões relacionadas às violações dos direitos humanos e à vulnerabilidade social.

Ao destacarem nas fotografias elementos que demonstram a degradação do ambiente, por exemplo, os EFs indicam a ausência de manutenção e cuidado nos espaços de convivência coletiva, simbolizando a negligência com áreas que são ocupadas, geralmente, por populações vulneráveis. Esse cenário de abandono pode ser entendido como um reflexo da marginalização enfrentada por essas comunidades, que muitas vezes vivem sem acesso a serviços básicos e infraestrutura de qualidade.

Em uma outra perspectiva, as fotografias que incluem figuras humanas, apesar de retratarem cenas cotidianas, também revelam camadas de vulnerabilidade social e violações dos direitos humanos quando observadas em profundidade (Figura 4). Essas imagens capturam não apenas as ações imediatas dos indivíduos, mas sugerem contextos de precariedade e desamparo que transcendem o óbvio, remetendo a questões estruturais e à ausência de garantias básicas.

**Figura 4:** Fotografias de cenas cotidianas de vulnerabilidade social – Brasília, DF, Brasil, 2025.

A cena de indivíduos aguardando transporte público, por exemplo, pode ser interpretada como um símbolo da escassez de acesso a um sistema de transporte eficiente, seguro e em quantidade adequada, evidenciando uma violação indireta ao direito à mobilidade e à liberdade de movimento^(7)^. Essa limitação não apenas dificulta a locomoção, mas também reflete a falta de infraestrutura urbana necessária para garantir a dignidade e o bem-estar das comunidades.

Outro simbolismo apresentado nas fotografias é a representação de pessoas carregando sacolas de lixo, ato que além de destacar a relação dos moradores com a gestão de resíduos, traz a reflexão do lixo como fonte de alimento. Tal realidade reflete o abandono dos direitos fundamentais, além de ilustrar a desumanização e insegurança alimentar, que são violações do direito a um meio ambiente saudável, à vida e à dignidade^(7)^.

Na interpretação iconográfica, foi possível perceber desigualdade e exclusão social nas imagens dos EFs antes mesmo de identificar os artigos violados da DUDH^(7)^. As composições fotográficas, elaboradas com atenção por eles, aliadas ao conhecimento dos artigos da declaração, tema abordado nas etapas iniciais da oficina, facilitaram a identificação dos simbolismos presentes nas imagens e o mapeamento preliminar dos direitos violados antes mesmo de serem apresentados em suas narrativas.

### Interpretação Iconológica e a Bioética Narrativa

A interpretação iconológica vai além da simples descrição visual e simbólica das imagens; ela buscou explorar as ideias, valores e significados profundos dos elementos dentro do contexto histórico e social em que a obra foi criada^(19,20)^. A bioética narrativa, por sua vez, complementou essa análise ao enriquecer a compreensão de obras que tratam de temas como vida e condição humana, proporcionando uma visão mais profunda das questões éticas e emocionais representadas^(13,14)^.

No caso das fotografias capturadas pelos EFs, essa abordagem integrada possibilitou a exploração não apenas dos aspectos visuais e técnicos das imagens, mas também das narrativas pessoais e coletivas que elas carregam. No contexto bioético, essas violações desafiam os princípios do principialismo, comprometendo a autonomia, justiça e beneficência no cuidado em saúde e na dignidade humana^(11,12)^. A bioética narrativa complementa essa perspectiva ao transformar as imagens em narrativas visuais que expõem essas desigualdades e dilemas éticos^(13,14)^. Esse processo estimula a empatia e a reflexão crítica, auxiliando os estudantes a compreender o impacto das violações de direitos humanos na assistência em enfermagem e reforçando a importância de uma prática pautada na ética, equidade e dignidade humana.

A inclusão dessas emoções no registro influenciou diretamente o impacto que cada estudante desejou transmitir ao seu observador. Os elementos “táteis” da fotografia e o simbolismo presente na composição das imagens se entrelaçaram com as ideias dos direitos humanos e dos princípios bioéticos, moldando a percepção e interpretação de todos os atores que as analisaram. Dessa forma, cada análise foi enriquecida por esses elementos, que, juntos, ampliaram a experiência e a compreensão das obras imagéticas.

Ao discutirem as emoções e reflexões surgidas durante a captura, os EFs puderam articular como essas imagens representam fragmentos das violações cotidianas em nossa sociedade. Suas verbalizações enriqueceram a compreensão das fotografias e dos artigos que elas evocam, promovendo uma discussão que incluiu as percepções dos EOs, que não fizeram registros, mas que conseguiram identificar outras violações menos evidentes para o fotógrafo.

Durante as análises, os EFs ressaltaram que, frequentemente, não percebem as violações de direitos que ocorrem diariamente com as pessoas ao seu redor, devido à normalização dessas situações em diversos contextos. Esse aspecto também foi destacado pelos EOs, que reconheceram não perceber com frequência as violações ao seu redor de maneira tão vívida como nas fotografias. Muitos desses estudantes relataram, inclusive, por não perceberem violações tão explícitas em seus contextos, que optaram por não enviar suas próprias imagens:

*Tive interesse, mas não tive tempo para buscar algo além do que já estava sendo fotografado por outros colegas. No entanto, percebo que, independentemente de quão explícitas sejam, as violações estão sempre presentes ao nosso redor!* (EO29).

Os EOs não apenas reconheceram e compreenderam o simbolismo por trás das fotografias, mas também ampliaram as interpretações, trazendo novas perspectivas que não haviam sido mencionadas anteriormente. Esse diálogo demonstra que houve compreensão mais profunda das questões sociais em pauta, permitindo que todos os estudantes, independentemente de sua participação, engajassem nas questões levantadas pelas fotografias (Figura 5).

**Figura 5:** Fotografia 19 e as narrativas dos estudantes – Brasília, DF, Brasil, 2025.

Ao ser analisado o papel social das imagens, todos os estudantes apontaram que tanto as fotografias que registraram quanto aquelas apresentadas ao longo da oficina desempenham um papel fundamental como ferramentas de comunicação crítica e transformação social, em diferentes contextos. Para os estudantes, as imagens não são apenas representações da realidade, mas também instrumentos poderosos para provocar reflexão, gerar debate e questionar normas estabelecidas:

*É através da fotografia que podemos evidenciar realidades diferentes e avançarmos em discussões importantes para a sociedade* (EO32).

*A fotografia é mais do que a retratação de momentos importantes; pode ser uma forma de crítica e denúncia social, apontando direitos que são violados e realidades que precisam ser mudadas* (EO40).

No que diz respeito às fotografias que registram violações aos direitos previstos na DUDH^(7)^, observa-se seu grande potencial de sensibilizar o público, influenciar atitudes e até inspirar mudanças sociais significativas. Esse impacto é particularmente relevante para os estudantes, como apontaram, uma vez que são futuros profissionais da saúde e, portanto, estarão inseridos em contextos de trabalho vulneráveis ou em contato direto com populações em situação de risco.

Percebe-se que, para todos os estudantes, o registro de violações aos artigos da DUDH^(7)^, assim como a compreensão da fotografia como uma ferramenta de crítica social, não é algo impossível ou difícil de ser realizado, uma vez que situações de pobreza, exclusão social e negligência governamental estão constantemente presentes em nossa sociedade, e a visualização dessas violações pode fomentar o desenvolvimento do senso crítico. No entanto, estes ressaltam que a identificação dessas violações é mais clara com um conhecimento prévio dos conceitos teóricos, especialmente no contexto acadêmico:

*A partir da abordagem da temática sobre direitos humanos e bioética, sinto que meu olhar e o meu senso crítico melhorou com relação ao início da disciplina. Se fosse abordado com mais amplitude dentro da universidade, os alunos teriam uma formação mais humana* (EO35).

Esse embasamento permite uma leitura mais crítica e aprofundada da realidade, facilitando a percepção das desigualdades estruturais e dos direitos que estão sendo desrespeitados. No contexto da saúde, conforme apontado por eles, essa educação diferenciada é essencial, pois os preparariam para identificar e enfrentar as desigualdades no acesso a cuidados, assim como as discriminações que podem ocorrer nos sistemas de saúde. Ao integrar essa abordagem à formação em enfermagem, reforça-se a importância de uma prática baseada na equidade, no respeito aos direitos humanos e na promoção de uma assistência mais justa e inclusiva.

A seguir, na seção da discussão, serão abordadas as implicações da fotografia como ferramenta pedagógica na educação em direitos humanos, em articulação com a bioética narrativa e o principialismo bioético. Serão analisados seus impactos na reflexão crítica e ética, destacando as violações sociais e seus reflexos na prática de enfermagem.

## DISCUSSÃO

Susan Sontag (2004) argumenta que a fotografia transforma eventos em objetos consumíveis, criando narrativas limitadas e subjetivas da realidade. Ela vê as fotografias como construções que podem simplificar a complexidade do evento original, tornando a análise ativa do observador essencial para seu entendimento^(23)^. Corroborando a ideia, Roland Barthes^(10)^ apresenta a fotografia como uma forma de narrativa que documenta e cria uma experiência pessoal.

A união das ideias de Sontag e Barthes permite uma reflexão profunda sobre imagem, realidade e interpretação. Sontag vê a fotografia como uma forma de consumo que cria narrativas visuais restritas, enquanto Barthes realça sua dimensão íntima, na qual o contexto e o detalhe emocional criam uma experiência única, transformando a relação entre o fotógrafo e o observador^(10,23)^. Assim, a fotografia, como ferramenta de transformação, alinha-se aos conceitos da bioética narrativa, pois não apenas registra uma realidade, mas também molda respostas éticas e emocionais únicas.

A bioética narrativa valoriza essa capacidade de sentir e interpretar experiências alheias, permitindo que a fotografia transcenda o papel de espelho da realidade, tornando-se um espaço de diálogo e empatia em questões humanas^(13,14)^. Aplicando a análise iconológica de Erwin Panofsky, que examina as imagens em seus contextos históricos e culturais, percebe-se que elas desvendam camadas profundas de significado. Essas camadas são cruciais para fomentar empatia e compreensão ética, enriquecendo tanto a experiência de quem registra quanto a de quem observa a fotografia^(19,20)^.

Esses conceitos ajudam a compreender como os estudantes de enfermagem que participaram da oficina percebem as violações dos direitos humanos em nossa sociedade, destacando a fotografia como crítica social. Estes entenderam que ela não apenas documenta, mas engaja e provoca empatia, abordando a injustiça de maneira que articula discursos sobre a humanidade e suas vulnerabilidades, funcionando como um elo entre o presente e a transformação social^(1,3–6)^.

Sob a ótica do principialismo bioético, essa abordagem reforça nos estudantes de enfermagem a importância dos princípios da autonomia, justiça, beneficência e não maleficência na saúde. A fotografia, ao expor desigualdades e estimular a reflexão crítica, amplia a percepção da responsabilidade ética na defesa dos direitos humanos. Assim, a análise imagética sensibiliza para os desafios das populações vulneráveis, fortalecendo a necessidade de uma atuação profissional pautada na equidade, respeito e bem-estar coletivo^(11,12)^.

Já no formato de imagem estática, a fotografia representa uma realidade próxima, mas, como aponta Secco^(2)^, permanece sujeita a influências externas tanto do fotógrafo quanto do observador. Quando enriquecida pelas narrativas, a fotografia transcende seu papel documental e pode se tornar um essencial instrumento acadêmico para promoção de debates e reflexões profundas sobre questões sociais e violações de direitos humanos, temas que estão interligados às práticas diárias da enfermagem. Além disso, essa abordagem fortalece a formação crítica dos estudantes, incentivando uma assistência mais humanizada e eticamente comprometida com a equidade e dignidade dos pacientes^(11,12)^.

Assim, o encontro entre bioética narrativa e fotografia ocorre ao empregar as narrativas e experiências dos estudantes de enfermagem na captura de imagens como instrumentos para estimular uma reflexão crítica e deliberação ética nos cuidados em saúde. Esse processo de integrar a narrativa com o ato fotográfico no ensino ultrapassa a simples transmissão de conhecimento, engajando os estudantes de enfermagem em uma análise profunda e ativa de questões éticas e sociais, promovendo uma compreensão mais holística e responsável das complexidades envolvidas na prática assistencial^(13,14)^.

Estudos com participantes de diferentes áreas e níveis de ensino mostram que a fotografia é uma ferramenta eficaz para conscientizar sobre questões sensíveis, construindo narrativas imagéticas que desconstroem práticas mecanicistas e ampliam o entendimento crítico^(10,19,23–25)^. Dessa forma, essa abordagem fortalece a formação dos estudantes, capacitando-os a identificar violações de direitos humanos e refletir sobre as condutas éticas a serem adotadas na prática profissional da enfermagem^(15#x2013;18,25)^. Além disso, promove um cuidado mais humanizado, reafirmando o compromisso da profissão com a justiça social, os princípios éticos e a dignidade dos pacientes^(11,12)^. No que tange à educação superior, é vital criar uma cultura de direitos humanos, conforme apontam as DCNs e a Política Nacional de Educação em Direitos Humanos^(15,26)^, que seja essencial para atitudes de defesa dos mais vulneráveis. Esta educação deve oferecer não apenas conhecimento técnico, mas também desenvolver habilidades críticas e éticas, preparando os futuros profissionais para atuarem com responsabilidade e reflexão^(17,26–28)^.

Integrar a educação em direitos humanos, em alinhamento aos princípios éticos, ao currículo prepara o indivíduo como um agente de mudança aberto ao diálogo e comprometido com a dignidade e equidade^(11,12,17,18,27,28)^. Para que esse aprendizado seja eficaz, é essencial que a educação vá além da teoria, utilizando atividades práticas como a fotografia. Esse método não só estimula uma reflexão profunda sobre as implicações éticas e sociais da prática profissional, mas também motiva os estudantes a engajar ativamente em atividades interativas, promovendo um aprendizado envolvente e crítico^(20,27,28)^.

Logo, percebe-se que os estudantes de saúde, principalmente os de enfermagem, devem receber formação técnica e conteúdo que integrem teoria e prática assistencial, permitindo a inserção em rotinas e protocolos de instituições de saúde^(25#x2013;28)^. A inclusão de temas transversais aos direitos humanos ajuda a formar profissionais aptos e conscientes para atuar com ética e respeito à dignidade e vulnerabilidades dos pacientes.

Apesar da pequena amostra e da aplicação em um único curso, a principal lacuna deste estudo foi a ausência de fotografias que representassem uma maior diversidade de violações dos direitos humanos. Essa limitação possivelmente decorre da baixa familiaridade dos acadêmicos de enfermagem com a temática dos direitos humanos ao longo da graduação, o que pode ter resultado na invisibilização de violações presentes em seu cotidiano. Esse cenário evidencia a necessidade de ampliar a abordagem sobre direitos humanos na formação em enfermagem, integrando a DUDH de maneira interdisciplinar ao currículo teórico e prático. A inclusão desse tema de forma mais estruturada não apenas contribuiria para o desenvolvimento de uma visão crítica e reflexiva dos estudantes, mas também fortaleceria sua atuação profissional no reconhecimento e enfrentamento das injustiças sociais e desigualdades em saúde.

## CONCLUSÃO

A oficina pedagógica desenvolvida neste estudo foi uma experiência significativa, permitindo que os estudantes se envolvessem tanto na captura quanto na análise crítica das imagens. O uso das fotografias como ferramenta didática para o ensino de direitos humanos estimulou a reflexão sobre questões sociais e direitos humanos, ampliando a compreensão dos participantes sobre as implicações éticas em sua futura prática profissional.

Além disso, a presente experiência pedagógica pode ser utilizada como ferramenta para identificar as fragilidades dos alunos no que tange à percepção e compreensão sobre as violações do DUDH, permitindo ao docente aprofundar e expandir o debate. A ausência de uma abordagem integrada entre bioética, enfermagem e direitos humanos limita a formação crítica e ética dos enfermeiros.

Logo, a associação entre práticas pedagógicas, como bioética narrativa, principialismo, direitos humanos e uso da fotografia, como ferramenta metodológica para integração e aplicação desses referenciais foi essencial. Tal fato contribuiu para o preparo de futuros profissionais empáticos, sensíveis às violações, com consciência ética sobre a dignidade e as vulnerabilidades diante das violações da DUDH, vivenciadas no cotidiano profissional da enfermagem.

## Data Availability

O conjunto de dados que dá suporte aos resultados deste estudo não está disponível publicamente devido à presença de informações sensíveis e à necessidade de preservação da confidencialidade dos participantes. As fotografias e demais materiais produzidos durante a oficina pedagógica estão armazenados em repositório acadêmico institucional de uso restrito à equipe de pesquisa, conforme aprovado pelo Comitê de Ética em Pesquisa da Faculdade de Ciências da Saúde da Universidade de Brasília (UnB) (Parecer Consubstanciado nº 6.312.687, de 20/09/2023). Todas as informações relevantes para a compreensão dos achados estão descritas no corpo do artigo.

# Translation: Bioética y derechos humanos en la formación de enfermería: relato de una experiencia pedagógica*

**DOI:** 10.1590/1980-220X-REEUSP-2024-0384es

**Authors:** Alessandra Lima Fontenele, Gabriele Pereira de Sena, Simone Luzia Fidelis de Oliveira, Dirce Bellezi Guilhem

**Affiliations:** 1 Universidade de Brasília, Faculdade de Ciências da Saúde, Programa de Pós-Graduação em Ciências da Saúde, Brasília, DF, Brasil.; 2 Fundação de Ensino e Pesquisa em Ciências da Saúde, Programa de Residência Uniprofissional de Enfermagem em Centro Cirúrgico. Brasília, DF, Brasil.; 3 Universidade de Brasília, Faculdade de Ciências da Saúde, Departamento de Enfermagem. Brasília, DF, Brasil.

**Keywords:** Bioética, Derechos Humanos, Enseñanza, Estudiantes de Enfermería, Fotografía

## Abstract

**Objetivo:**

Describir una experiencia pedagógica con estudiantes de enfermería que utilizaron la fotografía como herramienta didáctica para la enseñanza de los derechos humanos.

**Método:**

Se trata de un relato de experiencia pedagógica de estudio cualitativo transversal basado en la bioética narrativa, que utiliza las narrativas personales como instrumento analítico para profundizar en la comprensión de los problemas éticos, y en el análisis iconográfico, que investiga los significados y contextos profundos que subyacen a las fotografías registradas.

**Resultados:**

Las fotografías realizadas por los “estudiantes fotógrafos” retratan situaciones reales de la vida cotidiana de nuestra sociedad, resaltando temas como la pobreza, la exclusión social y la negligencia gubernamental, que, según los propios participantes, evocan violaciones de varios artículos de la Declaración Universal de Derechos Humanos.

**Consideraciones finales:**

El taller pedagógico utilizó la fotografía para involucrar a los estudiantes en reflexiones sobre derechos humanos y cuestiones sociales, promoviendo una formación ética y humanizada. A pesar de las limitaciones, el estudio reveló un déficit en la formación universitaria en derechos humanos, pero destacó el potencial de metodologías innovadoras para fortalecer la preparación crítica y empática de los futuros profesionales.

## INTRODUCCIÓN

La fotografía es una poderosa herramienta para el conocimiento histórico, representando acontecimientos y grupos sociales, y actuando como recurso pedagógico para analizar temas a menudo olvidados. Como experiencia social, la fotografía permite interpretaciones variadas, influidas por la herencia cultural de cada individuo. También permite analizar los discursos que conforman sus interpretaciones a partir de los contextos en los que fueron creadas^(1,2)^.

A través de la fotografía, los recuerdos y las emociones se eternizan, permitiendo plantear cuestiones sociales de forma democrática^(2#x2013;4)^. En el ámbito de los derechos humanos, la fotografía denuncia las violaciones y las luchas por la dignidad, inmortalizando acontecimientos como las guerras y la conquista de derechos^(5)^. Estas imágenes contrastan con la narrativa de derechos inalienables de las declaraciones, mostrando un mundo con innumerables violaciones^(5#x2013;7)^.

Así, la fotografía no sólo refleja la realidad, sino que también crea narrativas que fomentan la reflexión crítica. En la enseñanza de los derechos humanos, permite debates sobre el respeto y el cumplimiento de estos derechos, especialmente entre los futuros profesionales de la salud^(1,8,9)^. Al captar una imagen, el fotógrafo construye un discurso al decidir qué mostrar, reflejando una interpretación subjetiva de la realidad^(9)^. Barthes^(10)^ considera al fotógrafo como un narrador, cuyas elecciones influyen en la percepción del observador.

Esta relación entre imagen e interpretación refleja la construcción histórica de la dignidad humana, principio central de los derechos humanos y de la bioética, que se consolida como campo interdisciplinar que estudia la vida y sus dimensiones a la luz de valores y principios morales^(11,12)^. En este contexto, el principialismo bioético, basado en los principios de autonomía, beneficencia, no maleficencia y justicia, refuerza la necesidad de garantizar la equidad y el respeto a la dignidad humana, valores también garantizados por la Declaración Universal de los Derechos Humanos (DUDH)^(7,11,12)^.

La bioética narrativa, un enfoque emergente de la bioética y desarrollado por Tomás Moratalla^(13)^, complementa esta perspectiva articulando los principios morales con historias individuales y colectivas que valoran la experiencia y el contexto de cada individuo en la toma de decisiones éticas. En la enseñanza de los derechos humanos, este enfoque, combinado con imágenes, se convierte en una herramienta de sensibilización y análisis crítico, propiciando reflexiones sobre la dignidad, la justicia, la equidad y los principios bioéticos, contribuyendo a la formación de ciudadanos y profesionales de la salud conscientes y comprometidos con la ética y los derechos humanos^(11#x2013;14)^.

Valores como la dignidad y el respeto a los derechos humanos son fundamentales para una práctica de enfermería ética y humanizada. Las Directrices Curriculares Nacionales (DCN) para los cursos de enfermería de pregrado hacen hincapié en la necesidad de una formación humanística, crítica y reflexiva, que prepare a las enfermeras para actuar con responsabilidad social y compromiso ciudadano^(15)^. Estas directrices destacan la importancia de incluir contenidos relacionados con los derechos humanos, la ética y la bioética, que permitan a los profesionales identificar, afrontar y prevenir las desigualdades y la discriminación en salud^(16)^.

La inclusión de los derechos humanos y la bioética en la formación de enfermería garantiza unos cuidados basados en el respeto a la autonomía, la intimidad y la dignidad de los pacientes, principios fundamentales sustentados en el principialismo bioético y enriquecidos por la bioética narrativa^(11#x2013;14)^. De este modo, la formación no se limita al desarrollo técnico-científico, sino que refuerza el compromiso ético, reflexivo y humanizado de la profesión, en línea con los principios de la DUDH^(7,16)^.

Aunque el lenguaje de los derechos humanos aún no sea ampliamente utilizado en el día a día de la profesión, los principios establecidos en el Código Deontológico de los Profesionales de Enfermería, como el bienestar del paciente y de la comunidad, el respeto, la tolerancia y la honestidad, reflejan directamente los derechos garantizados por la DUDH, demostrando la intersección entre la ética profesional y los valores fundamentales de la bioética y la justicia social^(7,17,18)^.

Por lo tanto, teniendo en cuenta lo anterior, este estudio tuvo como objetivo describir una experiencia pedagógica con estudiantes de enfermería que utilizaron fotografías como herramienta didáctica para la enseñanza de los derechos humanos. Este abordaje buscó fortalecer el compromiso de los futuros profesionales con la construcción de una práctica más ética y humana, en consonancia con los principios éticos y de derechos humanos fundamentales.

## MÉTODO

### Tipo de Estudio

Se trata del relato de una experiencia de enseñanza, con abordaje cualitativo y transversal, realizada durante una pasantía de docencia doctoral en un programa de posgrado en ciencias de la salud de una universidad pública federal de Brasil. La base teórico-metodológica del estudio se basó en la bioética narrativa y en el análisis iconográfico, permitiendo un análisis más sensible y contextualizado de las violaciones de los principios de la DUDH^(7,13,14,19,20)^. Para garantizar el rigor metodológico, el estudio siguió los *COnsolidated* criteria *for REporting Qualitative research* ^(21)^.

### Lugar y Población del Estudio

El estudio se realizó en una universidad pública federal de Brasil, entre marzo y julio de 2024, con alumnos del 5º semestre del curso de pregrado en enfermería, matriculados en la asignatura Bioética y Legislación en Enfermería (BLE). Un total de 48 alumnos fueron invitados a participar en el «Taller Pedagógico Fotográfico», actividad propuesta como parte de la asignatura. El plan docente de BLE hace hincapié en la necesidad de abordar el tema de los derechos humanos y su importancia tanto para la asistencia sanitaria como para la investigación. Por lo tanto, la incorporación de la fotografía como herramienta pedagógica para la reflexión crítica permitió a los estudiantes explorar la intersección entre la teoría y la práctica de una manera diferente, estimulando las habilidades artísticas y la conciencia de las cuestiones bioéticas en la salud, reforzando su relevancia para la práctica profesional.

### Recolección de Datos

El «Taller Pedagógico Fotográfico» se organizó en cuatro etapas, todas diseñadas para no comprometer el progreso del curso de BLE. Fue planeado en conjunto con los profesores responsables, para garantizar que las actividades estuvieran en armonía con el cronograma académico del semestre.

Las dos primeras etapas consistieron en clases teóricas que abordaron los conceptos fundamentales, la historia y las directrices de los derechos humanos, así como el papel de la fotografía en la defensa de estos derechos, destacándola como herramienta de denuncia y transformación social. En ellas se abordó el tema de los derechos humanos, recogido en el plan docente de la asignatura, proporcionando a los alumnos una sólida base teórica y una comprensión crítica que les preparó para los momentos posteriores.

La tercera parte era una actividad práctica voluntaria, que consistía en enviar una fotografía inédita que ilustrase una violación de un artículo de la DUDH^(7)^. Las violaciones podían abarcar temas como la integridad (física, psicológica o patrimonial), la libertad, la seguridad, los derechos sociales, civiles y políticos, la vida y las violaciones de los derechos medioambientales. Sin embargo, la especificación del tipo de violación dependería del registro elegido por el alumno, lo que significa que no todas las formas de violación podrían cubrirse en el ejercicio^(7)^.

Se indicó a los alumnos que capturaran las imágenes (en color o en blanco y negro) con teléfonos móviles, debido a su facilidad de uso. A los alumnos que optaron por incluir a personas en las imágenes, se les facilitaron dos copias del Acuerdo para el uso de imágenes con fines científicos: una para el modelo y otra para entregar a los profesores para que la guardaran. Se les indicó que colgaran sus fotografías, con un título, el modelo de teléfono móvil utilizado y un pie de foto que incluyera el artículo de la DUDH^(7)^ violado (criterio para incluir las imágenes en el estudio), en la sala virtual de la asignatura vinculada a la matrícula y al correo electrónico institucional de la universidad. Sólo los profesores y el equipo de investigación podían gestionar el contenido de la sala virtual, lo que garantizaba la confidencialidad y la organización de las imágenes. Además, esta actividad era opcional y no evaluable, lo que garantizaba que su no realización no conllevaría sanciones académicas.

Por último, la cuarta parte del taller se dedicó a la discusión en clase de las fotografías tomadas por los alumnos. Durante esta fase, se presentaron las imágenes junto con su análisis iconográfico. Esto permitió a todos los alumnos analizar críticamente las fotografías tomadas y los artículos que habían sido violados, con el fin de poder analizarlos críticamente más adelante.

### Análisis y Procesamiento de Datos

En este estudio, el análisis de datos se basó en el análisis iconográfico de Panofsky^(19)^ y en la bioética narrativa de Tomás Moratalla^(13)^. El enfoque de Panofsky implicó tres etapas: preiconográfica, que examina los elementos visuales de las imágenes; iconográfica, que interpreta los significados y símbolos contenidos en ellas; e iconológica, que contextualiza los elementos en su entorno histórico y cultural. La bioética narrativa ha complementado este enfoque centrándose en las experiencias subyacentes a las imágenes, ayudando a interpretar las narraciones y las conexiones con las violaciones observadas de la DUDH^(7,13,14,19,20)^.

La perspectiva de Tomás Moratalla^(13)^, aplicada en este estudio, destaca las narrativas como herramienta pedagógica en bioética, permitiendo a los estudiantes comprender los dilemas éticos a través de experiencias concretas e historias representadas en las imágenes. Al integrar el análisis narrativo con los desafíos éticos de la práctica profesional, este enfoque fortaleció la conexión entre la bioética, los derechos humanos y la realidad social, preparando a los estudiantes para hacer frente a situaciones complejas en la asistencia sanitaria^(11#x2013;14)^.

### Aspectos Éticos

Este estudio siguió los principios éticos de la Resolución 466/2012^(22)^ del Consejo Nacional de Salud, y sólo se inició después de la aprobación del Comité de Ética en Investigación de la Facultad de Ciencias de la Salud de la Universidad de Brasilia, Dictamen Consubstanciado n^º^ 6.312.687, 20/09/2023. Todos los estudiantes fueron informados sobre la investigación y aceptaron participar voluntariamente firmando el Formulario de Consentimiento Informado.

Para garantizar el anonimato, los participantes fueron identificados como «estudiante fotógrafo (EF)», seguido de un código numérico, asignado según el orden cronológico de recepción de las fotografías (por ejemplo: EF01, EF02, etc.). Los participantes que no enviaron fotografías se identificaron como «estudiantes observadores (EO)», seguidos de un código numérico que comienza con el número 29, en continuidad con la numeración de los participantes en el taller y sumando el número de alumnos matriculados en el curso (por ejemplo: EO29, EO30, etc.).

## RESULTADOS

Entre los alumnos matriculados en el curso BLE, 48 participaron en el estudio. De ellos, 28 enviaron fotografías para la actividad práctica del tercer momento. Sin embargo, dos imágenes fueron excluidas por no cumplir los criterios establecidos, resultando una muestra final de 26 fotografías para su análisis (interpretación preiconográfica e iconográfica). Los alumnos que no enviaron sus fotografías, optando por participar sólo en las exposiciones (primer y segundo momento) y en el debate del cuarto momento, compartieron sus narraciones sobre las imágenes de sus compañeros.

El análisis preliminar permitió categorizar las fotografías en tres temas: (1) *La basura como símbolo de interferencia*, representando los residuos como marca de abandono y negligencia; (2) *La urbanización como punto de reflexión social*, destacando las contradicciones entre modernización y marginación; y (3) *La persona como centro de desigualdades*, destacando las vulnerabilidades y el impacto de las injusticias sociales en los individuos. Estas imágenes se organizaron según el orden cronológico en que se recibieron, lo que justifica la falta de una secuencia lineal dentro de las categorías.

Como se ilustra en la Figura 1, las fotografías captadas por los EFs muestran la pobreza, la exclusión social y la negligencia gubernamental, poniendo de relieve diversas violaciones de los derechos humanos. Tras un análisis preliminar, se observó que predominaban las violaciones relacionadas con la libertad, los derechos sociales, civiles y políticos, el derecho a la vida y el medio ambiente, tal y como se establece en la DUDH^(7)^.

**Figura 1:** Recopilación de las fotografías tomadas por los alumnos durante la actividad práctica - Brasilia, DF, Brasil, 2025.

### Análisis Preiconográfico

El análisis preliminar de las fotografías se centró en la observación objetiva de los elementos visibles y recurrentes en las imágenes, como las formas, los colores, las figuras y la composición de los elementos, sin asignar interpretaciones ni significados^(19,20)^. Como resultado, fue posible identificar la presencia constante de elementos degradantes en el entorno, como basura, escombros, grafiti y vegetación alta. En algunas de estas imágenes se registraron refugios improvisados, situados en zonas inadecuadas y expuestas a riesgos (Figura 2).

**Figura 2:** Elementos de degradación observados en las fotografías – Brasilia, DF, Brasil, 2025.

Por otro lado, aunque con menor frecuencia, algunas fotografías revelaron figuras humanas en situaciones cotidianas, como individuos esperando el transporte público, sosteniendo bolsas de basura y recibiendo asistencia sanitaria (Figura 3). También destaca la composición fotográfica de estas imágenes, con colores más fríos, incluso en blanco y negro, que contrastan con los colores cálidos predominantes en las demás fotografías. Otro aspecto destacable de la composición eran las líneas imaginarias que, «dibujadas» por los EFs durante la captura de la imagen, orientaban al espectador hacia los elementos destacados.

**Figura 3:** Figuras humanas vistas en las fotografías – Brasilia, DF, Brasil, 2025.

Esta variación estética y los elementos presentes en las fotografías enriquecieron la narrativa visual, proporcionando una comprensión más compleja y multifacética de la vida urbana vulnerable a las violaciones de la DUDH^(7)^, captada por los EFs. Además, esta diversidad estética contribuyó a estructurar la narrativa fotográfica, facilitando la interpretación iconográfica de las imágenes.

### Interpretación Iconográfica

La identificación iconográfica consistió en analizar las imágenes, asignando significados a los elementos visuales previamente identificados. El objetivo central de esta etapa era interpretar los símbolos, figuras y composiciones observados, situándolos en sus contextos sociales, religiosos, culturales o históricos^(19,20)^. En el caso de las fotografías tomadas por los EFs, la identificación iconográfica permitió explorar cómo los elementos visuales captados reflejan cuestiones relacionadas con la violación de los derechos humanos y la vulnerabilidad social.

Al destacar elementos en las fotografías que muestran la degradación del medio ambiente, por ejemplo, los EFs señalan la falta de mantenimiento y cuidado en espacios de convivencia colectiva, simbolizando el abandono de áreas generalmente ocupadas por poblaciones vulnerables. Este escenario de abandono puede entenderse como un reflejo de la marginación a la que se enfrentan estas comunidades, que a menudo viven sin acceso a servicios básicos e infraestructuras de calidad.

Desde otra perspectiva, las fotografías que incluyen figuras humanas, a pesar de representar escenas cotidianas, también revelan capas de vulnerabilidad social y violaciones de los derechos humanos cuando se observan en profundidad (Figura 4). Estas imágenes captan no sólo las acciones inmediatas de los individuos, sino que sugieren contextos de precariedad e indefensión que trascienden lo obvio, refiriéndose a cuestiones estructurales y a la ausencia de garantías básicas.

**Figura 4:** Fotografías de escenas cotidianas de vulnerabilidad social – Brasilia, DF, Brasil, 2025.

La escena de individuos esperando el transporte público, por ejemplo, puede interpretarse como un símbolo de la falta de acceso a un sistema de transporte eficiente, seguro y adecuado, mostrando una violación indirecta del derecho a la movilidad y a la libertad de circulación^(7)^. Esta limitación no sólo dificulta los desplazamientos, sino que también refleja la falta de infraestructuras urbanas necesarias para garantizar la dignidad y el bienestar de las comunidades.

Otro simbolismo presente en las fotografías es la representación de personas cargando bolsas de basura, acto que no sólo resalta la relación de los residentes con la gestión de residuos, sino que también reflexiona sobre la basura como fuente de alimento. Esta realidad refleja el abandono de los derechos fundamentales, además de ilustrar la deshumanización y la inseguridad alimentaria, que son violaciones del derecho a un medio ambiente sano, a la vida y a la dignidad^(7)^.

En la interpretación iconográfica, fue posible percibir la desigualdad y la exclusión social en las imágenes de los EFs incluso antes de identificar los artículos violados de la DUDH^(7)^. Sus cuidadas composiciones fotográficas, combinadas con el conocimiento de los artículos de la declaración, tema tratado en las fases iniciales del taller, facilitaron la identificación del simbolismo presente en las imágenes y el mapeo de los derechos vulnerados antes incluso de que fueran presentados en sus narrativas.

### Interpretación Iconológica y Bioética Narrativa

La interpretación iconológica va más allá de la simple descripción visual y simbólica de las imágenes; busca explorar las ideas, los valores y los significados profundos de los elementos dentro del contexto histórico y social en el que se creó la obra^(19,20)^. La bioética narrativa, a su vez, ha complementado este análisis enriqueciendo la comprensión de obras que abordan temas como la vida y la condición humana, proporcionando una visión más profunda de las cuestiones éticas y emocionales representadas^(13,14)^.

En el caso de las fotografías captadas por los EFs, este enfoque integrado permitió explorar no sólo los aspectos visuales y técnicos de las imágenes, sino también las narrativas personales y colectivas que portan. En el contexto bioético, estas violaciones desafían los principios del principialismo, poniendo en peligro la autonomía, la justicia y la beneficencia en salud y la dignidad humana^(11,12)^. La bioética narrativa complementa esta perspectiva transformando las imágenes en narraciones visuales que exponen estas desigualdades y dilemas éticos^(13,14)^. Este proceso estimula la empatía y la reflexión crítica, ayudando a los estudiantes a comprender el impacto de las violaciones de los derechos humanos en los cuidados de enfermería y reforzando la importancia de una práctica basada en la ética, la equidad y la dignidad humana.

La inclusión de estas emociones en el registro influyó directamente en el impacto que cada estudiante deseaba transmitir a su observador. Los elementos «táctiles» de la fotografía y el simbolismo presente en la composición de las imágenes se entrelazaron con las ideas de los derechos humanos y los principios bioéticos, moldeando la percepción e interpretación de todos los actores que las analizaron. De este modo, cada análisis se vio enriquecido por estos elementos, que en conjunto ampliaron la experiencia y la comprensión de las imágenes.

Al hablar de las emociones y reflexiones que surgieron durante la captura, los EFs pudieron articular cómo estas imágenes representan fragmentos de violaciones cotidianas en nuestra sociedad. Sus verbalizaciones enriquecieron la comprensión de las fotografías y de los artículos que evocan, promoviendo una discusión que incluía las percepciones de los EOs, que no realizaron los registros, pero que pudieron identificar otras violaciones menos evidentes para el fotógrafo.

Durante los análisis, los EFs señalaron que muchas veces no se dan cuenta de las violaciones de derechos que ocurren diariamente a las personas que los rodean, debido a la normalización de estas situaciones en diversos contextos. Este aspecto también fue destacado por los EOs, que reconocieron que no solían percibir las violaciones a su alrededor de forma tan vívida como en las fotografías. Muchos de estos estudiantes incluso afirmaron que, al no percibir violaciones tan explícitas en sus contextos, optaron por no enviar sus propias imágenes:

*Me interesaba, pero no tenía tiempo para buscar algo distinto a lo que ya estaban fotografiando otros compañeros. Sin embargo, me doy cuenta de que, por muy explícitas que sean, las violaciones siempre están presentes a nuestro alrededor»* (EO29).

Los EOs no sólo reconocieron y comprendieron el simbolismo que había detrás de las fotografías, sino que también ampliaron sus interpretaciones, aportando nuevas perspectivas que no se habían mencionado anteriormente. Este diálogo demuestra que había una comprensión más profunda de las cuestiones sociales en juego, lo que permitió a todos los alumnos, independientemente de su participación, comprometerse con las cuestiones planteadas por las fotografías (Figura 5).

**Figura 5:** Fotografía 19 y narraciones de los alumnos – Brasilia, DF, Brasil, 2025.

Al analizar el papel social de las imágenes, todos los alumnos señalaron que tanto las fotografías tomadas por ellos como las presentadas durante el taller desempeñan un papel fundamental como herramientas de comunicación crítica y transformación social en diferentes contextos. Para los alumnos, las imágenes no son sólo representaciones de la realidad, sino también poderosas herramientas para provocar la reflexión, generar debate y cuestionar las normas establecidas:

*Es a través de la fotografía como podemos poner de manifiesto diferentes realidades y avanzar en debates importantes para la sociedad* (EO32).

*La fotografía es más que retratar momentos importantes; puede ser una forma de crítica y denuncia social, señalando derechos que son violados y realidades que necesitan ser cambiadas* (EO40).

Con respecto a las fotografías que registran violaciones de los derechos establecidos en la DUDH^(7)^, existe un gran potencial para sensibilizar al público, influir en las actitudes e incluso inspirar un cambio social significativo. Este impacto es especialmente relevante para los estudiantes, como señalaron, ya que son futuros profesionales de la salud y, por lo tanto, trabajarán en contextos vulnerables o en contacto directo con poblaciones de riesgo.

Queda claro que, para todos los estudiantes, registrar violaciones a los artículos de la DUDH^(7)^, así como entender la fotografía como una herramienta de crítica social, no es algo imposible o difícil de lograr, ya que las situaciones de pobreza, exclusión social y abandono gubernamental están constantemente presentes en nuestra sociedad, y visualizar estas violaciones puede fomentar el desarrollo de un sentido crítico. Sin embargo, destacan que la identificación de estas violaciones es más clara con el conocimiento previo de conceptos teóricos, especialmente en el contexto académico:

Desde *que se abordó el tema de los derechos humanos y la bioética, siento que mi perspectiva y sentido crítico han mejorado en comparación con el inicio del curso. Si se abordara de forma más amplia dentro de la universidad, los alumnos tendrían una formación más humana* (EO35).

Este bagaje permite una lectura más crítica y profunda de la realidad, facilitando la percepción de las desigualdades estructurales y de los derechos que no se respetan. En el contexto sanitario, como señalaron, esta formación diferenciada es esencial, ya que les prepararía para identificar y afrontar las desigualdades en el acceso a los cuidados, así como las discriminaciones que pueden darse en los sistemas de salud. La integración de este enfoque en la formación de enfermería refuerza la importancia de una práctica basada en la equidad, el respeto a los derechos humanos y la promoción de cuidados más justos e inclusivos.

A continuación, en la sección de discusión, se abordarán las implicaciones de la fotografía como herramienta pedagógica en la educación en derechos humanos, en conjunción con la bioética narrativa y el principialismo bioético. Se analizará su impacto en la reflexión crítica y ética, destacando las violaciones sociales y su impacto en la práctica enfermera.

## DISCUSIÓN

Susan Sontag (2004) sostiene que la fotografía transforma los acontecimientos en objetos consumibles, creando narrativas limitadas y subjetivas de la realidad. Considera las fotografías como construcciones que pueden simplificar la complejidad del acontecimiento original, haciendo que el análisis activo del observador sea esencial para su comprensión^(23)^. Corroborando esta idea, Roland Barthes^(10)^ presenta la fotografía como una forma de narración que documenta y crea una experiencia personal^(10)^.

Combinar las ideas de Sontag y Barthes permite una profunda reflexión sobre la imagen, la realidad y la interpretación. Sontag ve la fotografía como una forma de consumo que crea narrativas visuales restringidas, mientras que Barthes enfatiza su dimensión íntima, en la que el contexto y el detalle emocional crean una experiencia única, transformando la relación entre el fotógrafo y el observador^(10,23)^. Así, la fotografía, como herramienta de transformación, se alinea con los conceptos de la bioética narrativa, ya que no sólo registra una realidad, sino que también moldea respuestas éticas y emocionales únicas.

La bioética narrativa valora esta capacidad de sentir e interpretar las experiencias ajenas, permitiendo que la fotografía trascienda su papel de espejo de la realidad, convirtiéndose en un espacio de diálogo y empatía sobre cuestiones humanas^(13,14)^. Aplicando el análisis iconológico de Erwin Panofsky, que examina las imágenes en sus contextos históricos y culturales, uno se da cuenta de que desvelan profundas capas de significado. Estas capas son cruciales para fomentar la empatía y la comprensión ética, enriqueciendo la experiencia tanto de la persona que graba como de la que observa la fotografía^(19,20)^.

Estos conceptos nos ayudan a entender cómo los estudiantes de enfermería que participaron en el taller perciben las violaciones de los derechos humanos en nuestra sociedad, destacando la fotografía como crítica social. Entendieron que no sólo documenta, sino que compromete y provoca empatía, abordando la injusticia de forma que articula discursos sobre la humanidad y sus vulnerabilidades, actuando como vínculo entre el presente y la transformación social^(1,3-6)^.

Desde la perspectiva del principialismo bioético, este enfoque refuerza en los estudiantes de enfermería la importancia de los principios de autonomía, justicia, beneficencia y no maleficencia en salud. Al exponer las desigualdades y estimular la reflexión crítica, la fotografía amplía la percepción de la responsabilidad ética en la defensa de los derechos humanos. Así, el análisis de imágenes sensibiliza sobre los desafíos que enfrentan las poblaciones vulnerables, fortaleciendo la necesidad de una actuación profesional basada en la equidad, el respeto y el bienestar colectivo^(11,12)^.

En el formato de imagen fija, la fotografía representa una realidad cercana, pero, como señala Secco (2021), permanece sujeta a influencias externas tanto del fotógrafo como del observador. Cuando se enriquece con narraciones, la fotografía trasciende su papel documental y puede convertirse en una herramienta académica esencial para promover debates y reflexiones en profundidad sobre cuestiones sociales y violaciones de los derechos humanos, temas que están interconectados con las prácticas diarias de enfermería. Además, este enfoque refuerza la formación crítica de los estudiantes, fomentando unos cuidados más humanizados y éticamente comprometidos con la equidad y la dignidad del paciente^(11,12)^.

Así, el encuentro entre la bioética narrativa y la fotografía se produce al utilizar las narrativas y experiencias de los estudiantes de enfermería en la captación de imágenes como herramientas para estimular la reflexión crítica y la deliberación ética en salud. Este proceso de integración de la narrativa con el acto fotográfico en la enseñanza va más allá de la simple transmisión de conocimientos, involucrando a los estudiantes de enfermería en un análisis profundo y activo de las cuestiones éticas y sociales, promoviendo una comprensión más holística y responsable de las complejidades implicadas en la práctica asistencial^(13,14)^.

Estudios con participantes de diferentes áreas y niveles de enseñanza demuestran que la fotografía es una herramienta eficaz para sensibilizar sobre temas sensibles, construyendo narrativas imaginativas que deconstruyen prácticas mecanicistas y amplían la comprensión crítica^(10,19,23–25)^. De este modo, este enfoque refuerza la formación de los estudiantes, permitiéndoles identificar las violaciones de los derechos humanos y reflexionar sobre los comportamientos éticos a adoptar en la práctica profesional enfermera^(15#x2013;18,25)^. También promueve cuidados más humanizados, reafirmando el compromiso de la profesión con la justicia social, los principios éticos y la dignidad del paciente^(11,12)^. En cuanto a la educación superior, es fundamental crear una cultura de derechos humanos, como se señala en las DCN y en la Política Nacional de Educación en Derechos Humanos^(15,26)^, que es esencial para las actitudes en defensa de los más vulnerables. Esta educación no sólo debe ofrecer conocimientos técnicos, sino también desarrollar habilidades críticas y éticas, preparando a los futuros profesionales para actuar de forma responsable y reflexiva^(17,26–28)^.

Integrar la educación en derechos humanos, en línea con los principios éticos, en el currículo prepara al individuo como agente de cambio abierto al diálogo y comprometido con la dignidad y la equidad^(11,12,17,18,27,28)^. Para que este aprendizaje sea eficaz, es esencial que la educación vaya más allá de la teoría, utilizando actividades prácticas como la fotografía. Este método no sólo fomenta la reflexión en profundidad sobre las implicaciones éticas y sociales de la práctica profesional, sino que también motiva a los estudiantes a participar activamente en actividades interactivas, promoviendo un aprendizaje participativo y crítico^(20,27,28)^.

Por lo tanto, es evidente que los estudiantes de salud, especialmente los de enfermería, deben recibir una formación técnica y contenidos que integren la teoría y la práctica asistencial, que les permita incluirse en las rutinas y protocolos de las instituciones sanitarias^(25#x2013;28)^. La inclusión de temas transversales de derechos humanos contribuye a formar profesionales capaces y conscientes de actuar con ética y respeto a la dignidad y vulnerabilidad de los pacientes.

A pesar de lo reducido de la muestra y de la aplicación a un único curso, la principal carencia de este estudio fue la ausencia de fotografías que representaran una mayor diversidad de violaciones de los derechos humanos. Esta limitación se debe posiblemente al desconocimiento del tema de los derechos humanos por parte de los estudiantes de enfermería durante sus estudios de grado, lo que puede haber provocado la invisibilización de violaciones presentes en su vida cotidiana. Este escenario pone de manifiesto la necesidad de ampliar el enfoque de los derechos humanos en la formación de enfermería, integrando la DUDH de forma interdisciplinar en el currículo teórico y práctico. La inclusión de este tema de forma más estructurada no sólo contribuiría al desarrollo de una visión crítica y reflexiva en los estudiantes, sino que también fortalecería su desempeño profesional en el reconocimiento y enfrentamiento de las injusticias y desigualdades sociales en salud.

## CONCLUSIÓN

El taller pedagógico desarrollado en este estudio fue una experiencia significativa, que permitió a los estudiantes involucrarse tanto en la captura como en el análisis crítico de las imágenes. El uso de fotografías como herramienta didáctica para la enseñanza de los derechos humanos estimuló la reflexión sobre temas sociales y derechos humanos, ampliando la comprensión de los participantes sobre las implicaciones éticas para su futura práctica profesional.

Además, esta experiencia pedagógica puede utilizarse como herramienta para identificar los puntos débiles de los alumnos en términos de percepción y comprensión de las violaciones de la DUDH, permitiendo a los profesores profundizar y ampliar el debate. La falta de un enfoque integrado entre bioética, enfermería y derechos humanos limita la formación crítica y ética de las enfermeras.

Por lo tanto, la asociación entre prácticas pedagógicas como la bioética narrativa, el principialismo, los derechos humanos y el uso de la fotografía como herramienta metodológica para integrar y aplicar estas referencias fue esencial. Esto ha contribuido a la preparación de futuros profesionales empáticos, sensibles a las violaciones y éticamente conscientes de la dignidad y las vulnerabilidades frente a las violaciones de la DUDH que se experimentan en la práctica diaria de enfermería.

## Data Availability

El conjunto de datos que respalda los resultados de este estudio no está disponible públicamente debido a la presencia de información sensible y a la necesidad de preservar la confidencialidad de los participantes. Las fotografías y demás materiales producidos durante el taller pedagógico están almacenados en un repositorio académico institucional de acceso restringido al equipo de investigación, conforme a la aprobación del Comité de Ética en Investigación de la Facultad de Ciencias de la Salud de la Universidad de Brasília (UnB) (Dictamen Consubstanciado n.^º^ 6.312.687, del 20/09/2023). Toda la información relevante para la comprensión de los hallazgos está descrita en el cuerpo del artículo.
